# A virtual reality system for delivery of military-specific vestibular rehabilitation after mild traumatic brain injury: the Praxis study protocol

**DOI:** 10.3389/fneur.2025.1558795

**Published:** 2025-05-21

**Authors:** Abdulmohsen M. Alroumi, Carrie W. Hoppes, Susan L. Whitney, Zhihao Li, Lisa Holt, Sridhar Ramakrishnan, Shannon L. Barnicott, Meghan T. Logeais, Holly Richard, Shane R. Salter, Jeffrey M. Tiede, Michael D. Wirt, Pedram Hovareshti

**Affiliations:** ^1^Department of Physical Therapy, University of Pittsburgh, Pittsburgh, PA, United States; ^2^Advanced Exposures, Diagnostics, Interventions, and Biosecurity (AEGIS) Program, Joint Base San Antonio-Lackland Air Force Base, San Antonio, TX, United States; ^3^BlueHalo, Germantown, Maryland, MD, United States; ^4^Center for the Intrepid, Department of Rehabilitation Medicine, Brooke Army Medical Center, Joint Base San Antonio-Fort Sam Houston, San Antonio, TX, United States; ^5^Department of Radiology, Brooke Army Medical Center, Joint Base San Antonio-Fort Sam Houston, San Antonio, TX, United States

**Keywords:** vestibular rehabilitation, mild traumatic brain injury, virtual reality, neurorehabilitation, military medicine, multisensory integration, neuroimaging biomarkers, return to duty

## Abstract

Mild traumatic brain injury (mTBI) is a prevalent issue among military service members, often resulting in persistent vestibular dysfunction that can impair operational performance. Vestibular, sensory, and cognitive deficits following mTBI hinder critical warrior tasks such as moving under fire and maintaining situational awareness. Despite advances in rehabilitation, there remains a need for interventions that replicate the complex demands of military environments. This study introduces Praxis, a novel virtual reality (VR) system designed to deliver multisensory vestibular rehabilitation to service members with persistent post-mTBI symptoms. Praxis integrates low-cost wearable sensors and VR environments to enhance gaze stability, balance, and cognitive-motor integration through military-relevant tasks. The primary goals of this pilot study are twofold: 1) to evaluate the feasibility of using the Praxis system to deliver VR, military-specific multisensory rehabilitation to a representative sample of 15 service members with vestibular-related complaints post-mTBI over a 4-week period, and 2) to evaluate objective measures, including advanced neuroimaging, to support return-to-duty (RTD) decisions by correlating functional performance improvements with neurophysiological changes. Patients will complete multisensory exercises using Praxis in conjunction with standard rehabilitation provided by the Special Operations Performance and Recovery (SPaR) Program at the Center for the Intrepid. A control group without vestibular-related complaints post-mTBI will receive the standard SPaR Program treatments. Outcome measures include Praxis scores, self-reported questionnaires, functional performance on military-specific tasks, self-reported symptom severity, and neurophysiological changes assessed through resting-state functional magnetic resonance imaging (rs-fMRI). Usability will be evaluated using the System Usability Scale (SUS), with success defined by a SUS score of 68 or higher. Secondary objectives explore the correlation between performance improvements, neurophysiological changes, and rehabilitation compliance. By capturing objective data through wearable sensors and advanced neuroimaging, this study aims to bridge the gap between clinical assessments and real-world functional performance. The findings will inform future large-scale trials and provide critical insights into the role of VR and multisensory rehabilitation in post-mTBI recovery, supporting improved RTD decisions and enhancing warfighter readiness. This protocol contributes to the advancement of neurorehabilitation by integrating technology-driven solutions tailored for military populations.

**Clinical trial registration:**

clinicaltrials.gov, identifier: NCT06314464.

## Introduction

Mild traumatic brain injury (mTBI) is a prevalent issue among military service members (SMs) (1), often resulting in persistent vestibular dysfunction that is increasingly linked to cognitive and proprioceptive impairments, which can further compromise a SM's operational capabilities (2–4). Advancing recovery strategies for SMs post-mTBI is paramount to ensuring military readiness and safety. Sensory, motor, and cognitive deficits following mTBI can significantly impair a SM's ability to perform critical warrior tasks, such as moving under fire and maintaining situational awareness, which are essential for combat effectiveness and survival (3, 5). Despite the availability of various rehabilitation techniques, there remains a critical need for interventions that not only address these multifaceted impairments but also translate into tangible improvements in operational performance. Current return-to-duty (RTD) decisions often rely on self-reported symptoms and standardized physical and cognitive tests that may not accurately reflect the functional demands of military activities (6–9). This highlights a significant gap in rehabilitation approaches: the lack of objective, ecologically valid tools that address both the physical and cognitive demands of military tasks while being accessible and portable for widespread use.

Current rehabilitation techniques, such as the Computer-Assisted Rehabilitation Environment (CAREN; Motek Medical, Houten, Netherlands) and tablet-based tools like VestAid (BlueHalo, Germantown, MD), have shown promise in addressing vestibular dysfunction. The CAREN is a large, virtual reality (VR) system that combines multi-planar motion with immersive virtual environments projected onto a curved, panoramic screen. It utilizes a motion capture system to track movement in real time and can be customized to stimulate the vestibular, somatosensory, and visual systems. Within the Military Health System, the CAREN has been successfully utilized to treat vestibular and balance deficits in SMs with mTBI (10). Moreover, performance on immersive balance tasks in the CAREN has been shown to distinguish SMs with comorbid mTBI and post-traumatic stress disorder (PTSD) from those with mTBI alone, suggesting that leveraging virtual environments in the CAREN may provide greater insight into vestibular complaints and more targeted interventions (11). However, the CAREN's large physical footprint, high cost, and need for a specialized engineer operator to build the scenarios and run the system limit its accessibility and scalability. VestAid is a tablet-based technology developed for vestibular rehabilitation, using the device's camera to track head and eye movements and provide real-time feedback for patients and clinicians (12). While VestAid is portable, it lacks the immersive, multisensory integration necessary for comprehensive rehabilitation. These limitations underscore the need for a novel rehabilitation system that is low-cost, portable, and capable of delivering immersive, ecologically valid rehabilitation tailored to military-specific tasks.

To address this gap, we developed Praxis (BlueHalo, Germantown, MD), a novel, portable system incorporating low-cost wearable sensors and a VR environment, designed to deliver effective multisensory rehabilitation exercises that hold military face validity. This innovative approach aims to bridge the gap between traditional rehabilitation techniques and real-world demands faced by SMs. Three VR scenarios were designed to enhance gaze stability, balance, and cognitive-motor integration. Unlike other VR-based systems, Praxis is uniquely designed to deliver dual-task and multisensory exercises that mirror the operational challenges faced by service members while remaining accessible and scalable for widespread use.

Research indicates that vestibular dysfunction post-mTBI corresponds with increased centrality in key brain regions including the frontal cortex, cingulate eye field nodes, and visual cortex (2, 4). These findings indicate that rehabilitation must encompass more than just physical recovery; it should also address cognitive, visual, and proprioceptive domains to facilitate comprehensive recovery. Targeted interventions that encourage SMs to shift from visual to proprioceptive feedback during tasks may accelerate recovery, as suggested by Smith et al. (4). Furthermore, evidence from Dieterich et al. demonstrate the brain's ability to reallocate resources in response to vestibular deficits, reinforcing the potential for targeted rehabilitation to effectively reprogram brain function (13). Resting-state functional magnetic resonance imaging (rs-fMRI) studies further emphasize the relationship between vestibular rehabilitation and neurophysiological improvements as seen in patients with vestibular migraine (14). Changes in spontaneous brain activity, measured by fractional amplitude of low-frequency fluctuation (fALFF), correlated with patient-reported improvements in dizziness and other impairments, suggesting that similar neurophysiological markers could be monitored in SMs undergoing multisensory rehabilitation for mTBI. Quantifying these changes and correlating them with functional improvements in real-world tasks, such as those encountered in military training and operations, could provide critical insights into the effectiveness of multisensory rehabilitation strategies (15, 16). Additionally, functional connectivity changes observed during recovery from mTBI further support the notion that targeted rehabilitation can induce measurable brain plasticity that corresponds with recovery (17, 18). By incorporating advanced neuroimaging techniques, such as rs-fMRI, this study aims to quantify neurophysiological changes associated with Praxis rehabilitation, providing possible evidence of neuroplasticity alongside objective measures of Praxis effectiveness in improving functional performance.

The primary goals of this pilot study are twofold: 1) to evaluate the feasibility of using the Praxis system to deliver VR, military-specific multisensory rehabilitation to a representative sample of SMs with vestibular-related complaints post-mTBI over a four-week period, and 2) to evaluate objective measures, including advanced neuroimaging, to support RTD decisions by correlating functional performance improvements with neurophysiological changes. This dual focus ensures that Praxis not only addresses the immediate rehabilitation needs of SMs but also provides measurable, objective data to guide RTD decisions. The inclusion of objective, robust, and sensitive assessments enabled by recent advances in wearable sensor technology (19–24) will be critical in determining the added value of the Praxis system in a military medicine context.

Our secondary aim is to explore the relationship between Praxis scores, self-reported questionnaires, functional performance on military-specific tasks, self-reported symptom severity, neurophysiological changes, and rehabilitation compliance. By analyzing these metrics, we aim to provide preliminary insights into the effectiveness of Praxis in detecting and influencing measurable changes in readiness performance during recovery from mTBI. The insights gained from this study could pave the way for larger trials, aimed at changing the approach of post-mTBI rehabilitation and ensuring that SMs are fully prepared for the demands of their warfighter duties upon return to active service. The findings from this study will lay the groundwork for larger trials, potentially enhancing post-mTBI rehabilitation and military readiness assessments.

## Methods

This is a prospective study of a Control and a Praxis group to determine if a novel multi-sensorimotor and vestibular training program results in enhanced functional outcomes in the military population. This study has been registered on ClinicalTrials.gov (NCT06314464). It was approved by the San Antonio Institutional Review Board (C.2023.055) and the U.S. Army Medical Research and Development Command, Office of Human Research Oversight. The patients/participants will provide their written informed consent to participate in this study.

## Participants

Fifteen SMs with a self-reported history of mTBI and unresolved vestibular-related complaints from the Center for the Intrepid's Special Operations Performance and Recovery (SPaR) Program at Brooke Army Medical Center will take part in a multisensory vestibular rehabilitation program. These participants will undergo a 4-week exercise program that includes gaze stabilization, dual-task balance exercises, spatial navigation, and agility training delivered via Praxis VR software in addition to the standard SPaR Program treatments. Another group of 15 SMs, who will not participate in the multisensory rehabilitation but will instead receive the standard SPaR Program treatments, will serve as the Control group. The Control group will consist of SMs who may or may not have a history of mTBI but do not report persistent vestibular-related complaints (i.e., dizziness or imbalance), allowing for comparison to individuals without vestibular dysfunction and providing normative data for self-reported symptom severity and functional performance on military-specific tasks in this unique population. All SPaR participants, including those in the Praxis group and the Control group, complete a comprehensive performance program that includes musculoskeletal injury rehabilitation, nutritional assessment, and psychological assessment and treatment. Recruitment efforts will focus on ensuring diversity in sex, ethnicity, and socioeconomic status to ensure that the sample is representative of and applicable to the broader military population.

### Inclusion criteria

Participants in the Praxis group will be those involved in the SPaR Program, aged 18–50 years, with either a self-reported or clinician-confirmed diagnosis of mTBI [as defined in the VA/DoD Clinical Practice Guideline for Management and Rehabilitation of Post-Acute mTBI (25)], and who continue to experience dizziness or imbalance. Additionally, they must be right-handed, as assessed by the Edinburgh Handedness Inventory-Short Form (26). As a pilot study with limited sample size, we chose to exclude left-handed subjects to reduce data variations. Given significant differences in functional brain localization between left- and right-handed individuals (27), particularly in motor (28) and language areas (29), such a focused inclusion can provide a more homogeneous sample, potentially increasing our statistical power. Additionally, this selection aligns with the technical constraints of our current VR system, which displays a right-handed firearm, and the hardware configuration, which includes a replica M4 rifle that houses only the right-hand VR controller. Presenting left-handed users with a right-handed physical and virtual weapon could create visuomotor discordance and disorientation, potentially compromising both the user experience and data quality. The Control group participants will also be from the SPaR Program, within the same age range, with or without a history of mTBI but without current complaints of dizziness or imbalance.

### Exclusion criteria

Participants will be excluded if they have any cognitive impairment (e.g., altered mental capacity due to administration of any mind-altering substances or significant stress/life circumstances, as assessed by standardized neuropsychological tests or clinician judgment), exhibit behavior that could compromise data collection or safety during the study, report severe pain during the evaluation (7/10 on a subjective pain scale), are pregnant (due to balance-related concerns; a pregnancy test will be administered for women of childbearing potential who are not using an acceptable form of contraception), or cannot refrain from alcohol or medications that might affect their balance or cerebral blood flow within 24 h prior to rs-fMRI and other repeat testing. Additionally, those who are mixed or left-handed as determined by the Edinburgh Handedness Inventory-Short Form (26), or those who cannot undergo MRI (e.g., due to non-MRI compatible devices, embedded metal fragments, or severe claustrophobia) for the Praxis group undergoing rs-fMRI will also be excluded. PTSD is not an exclusion criterion unless it results in cognitive impairment or unsafe behavior, ensuring that the sample remains representative of the broader military population with mTBI.

To ensure the Control group provides an adequate comparison, both groups will undergo the same baseline and follow-up assessments, including functional performance tests and self-reported symptom measures. This design allows for direct comparison of outcomes between SMs with and without persistent vestibular dysfunction, providing insights into the specific effects of vestibular rehabilitation. Furthermore, any group differences identified will inform future studies by highlighting the unique contributions of vestibular rehabilitation to functional recovery post-mTBI, potentially guiding clinical approaches for SMs with and without vestibular complaints.

## Study procedures

### Outlined study procedures

Following enrollment, each participant will be provided with an Oura ring™ (Oura Health Oy; San Francisco, CA) to wear throughout the study period. All participants will then complete the baseline Readiness Assessment Battery. Those in the Praxis group will also undergo a baseline rs-fMRI scan. Over the next 4 weeks, the Praxis group will participate in the SPaR Program combined with 45 min of supervised Praxis therapy daily, 5 days a week, in addition to a home exercise program (HEP) consisting of 20 min of unsupervised VestAid therapy daily, 5 days a week. VestAid is a tablet-based technology designed to support vestibular rehabilitation by monitoring and guiding patients through vestibulo-ocular reflex (VORx1) exercises (12). Participants in the Praxis group report progress at the end of each week (four times) using a Global Rating of Change (GROC) (30). The Control group participants will complete 4 weeks of the SPaR Program without a HEP. At the 4-week follow-up, all participants will retake the questionnaires and the Readiness Assessment Battery. Those in the Praxis group will undergo a follow-up rs-fMRI scan and will be asked to evaluate their experience with the Praxis system using the SUS. Over the course of the study, Praxis group participants will engage in approximately 20 sessions of Praxis and VestAid (5 days a week for 4 weeks).

### Detailed study procedures

#### Baseline assessment

Demographic information [including age, sex, height, weight, body mass index (BMI), Military Occupational Specialty/Area of Concentration (MOS/AOC), time in service, number and duration of deployments, number of self-reported and clinician-confirmed mTBI incidents and their causes, support structure (e.g., single, married), history of migraines/headaches, history of attention deficit hyperactivity disorder and/or learning disorders, and medical/surgical history] will be collected from all participants.

##### Questionnaires

The following self-reported questionnaires will be administered: the Dizziness Handicap Inventory (DHI) (31, 32), Pittsburgh Sleep Quality Index (PSQI) (33), Generalized Anxiety Disorder scale (GAD-7) (34), Post-Traumatic Stress Disorder Check List—Military Version (PCL-M) (35), Vestibular Activities Avoidance Instrument-9 (VAAI-9) (36), Headache Impact Test-6 (HIT-6) (37), perception of fogginess [assessed on a scale of 0 to 10 as per the Vestibular/Ocular Motor Screening (VOMS) (38)] to measure this symptom as a known indicator of multiple-domain adverse effects in athletes post-mTBI (39), and the Santa Barbara Sense of Direction Scale (SBSOD) (40). Participants will also rate their current functional level on a scale from 0 to 100, with 100 indicating optimal function (41).

##### Readiness Assessment Battery

All participants will undergo an assessment battery that includes the 4-Item Hybrid Assessment of Mobility for mild Traumatic Brain Injury (HAM-4-mTBI) (42), modified version of the Portable Warrior Test of Tactical Agility (POWAR-TOTAL) (43, 44), 5-10-5 Shuttle Run (45), and 300-yard Shuttle Run (45, 46) tests. Opal inertial sensors and Mobility Lab software (APDM™ wearable technologies, Portland, OR) will be used for all assessments to record objective outcomes. The Opal inertial sensors are worn on the forehead, sternum, lumbar spine, non-dominant wrist, and dorsum of each foot during the assessment battery. Opal-based outcome measures will include: peak lumbar turning speed (peak angular rate of the pelvis in the transverse plane); head turning speed (peak angular rate of the head in the transverse plane); upper trunk turning speed (peak angular rate of the upper trunk in the transverse plane); head-body coordination timing (peak angular displacement between the head and the trunk measured just before or at the onset of trunk axial rotation during turning, measured in the transverse plane); and head turn symmetry (left-to-right head turn velocity ratio).

###### HAM-4-mTBI

The HAM-4-mTBI (42) is a condensed, hybrid mobility assessment based on the Functional Gait Assessment (FGA) (47) and the High-Level Mobility Assessment Tool (HiMAT) (48) to monitor individuals with mTBI. Participants will complete the FGA gait with horizontal head turns and gait with pivot turn, as well as the HiMAT fast forward and backward walk. Outcomes measured from the HAM-4-mTBI will include the Opal-based measures plus the FGA gait with horizontal head turns and gait with pivot turn scored according to FGA criteria (scored 0 to 3) and the HiMAT fast forward and backward walk scored according to HiMAT criteria (middle 10 m of a 20 m trial is timed, scored 1 to 4).

###### Modified Version of the Portable Warrior Test of Tactical Agility (POWAR-TOTAL)

**Figure 1:**
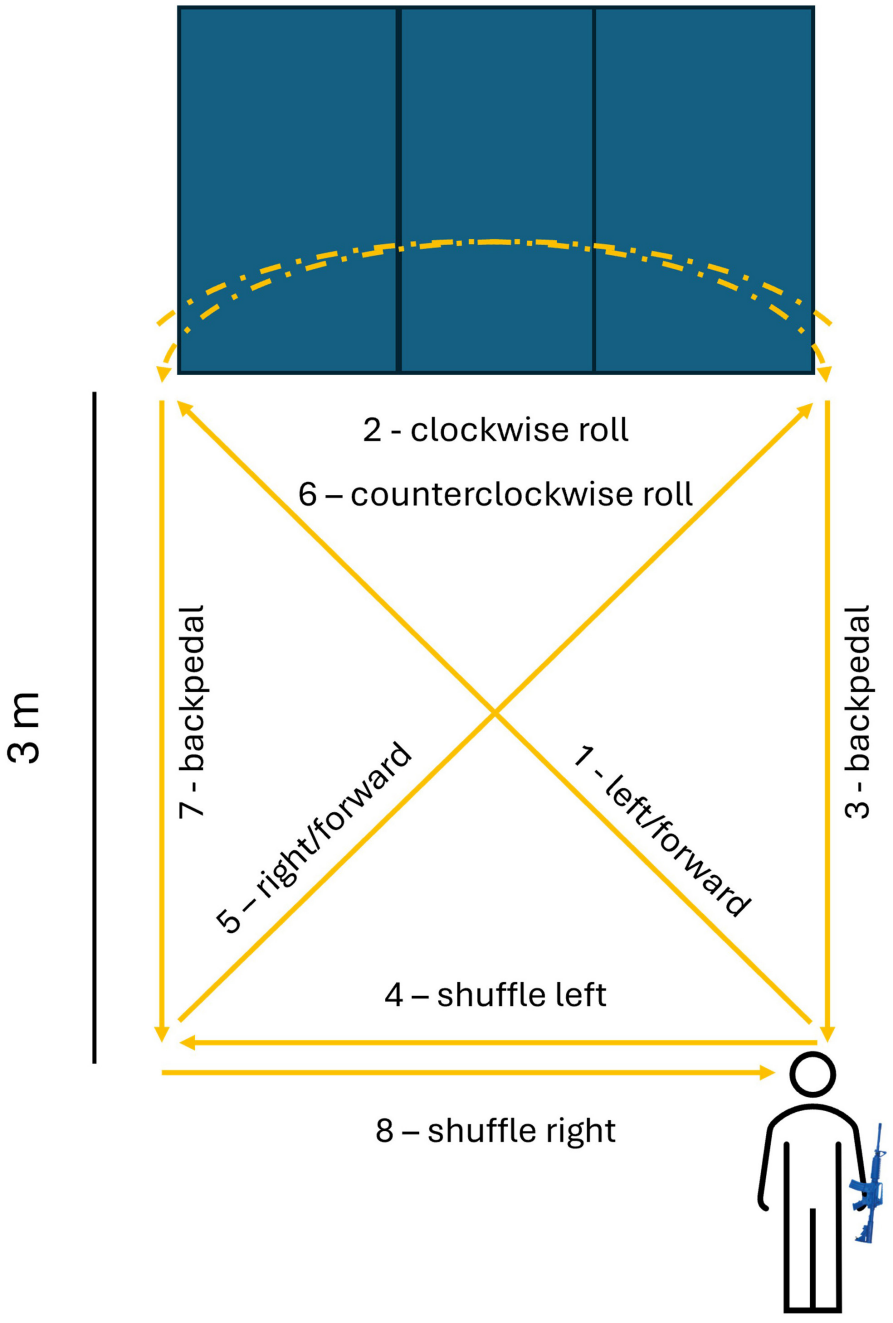
Schematic of the modified version of the Portable Warrior Test of Tactical Agility (POWAR-TOTAL). The service member begins the test in prone (pictogram) and follows the path direction of the (yellow) arrows. Combat rolls are performed on a folding tumbling mat (blue rectangle).

During the modified version of the POWER-TOTAL (43, 44), the participant will carry a simulated standard service weapon (Bluegun M4). The participant begins the test in a prone position (Figure 1). They will then stand up, run diagonally left/forward for 3 m, drop back into a prone position on a floor mat and perform a clockwise combat roll. Afterward, they will stand and backpedal to the starting position, side-shuffle to the left, run diagonally right/forward for 3 m, drop back into a prone position on a floor mat and perform a counterclockwise combat roll, stand up and backpedal to the starting line, and side-shuffle to the right to end at the starting position. The time taken to complete the single-task modified version of the POWAR-TOTAL will be used to determine the interval for the cognitive-only single-task. Specifically, the participant is told an 8-digit grid coordinate, the stopwatch is started immediately after the final digit is spoken, the duration equivalent to the single-task POWAR-TOTAL completion time is waited, and then the participant is asked to recall the grid coordinate. Then, this cognitive task is combined with the physical task for a cognitive dual-task paradigm where the participant must remember an 8-digit grid coordinate; immediately upon completion of the modified version of the POWAR-TOTAL the participant will be asked to recall the 8-digit grid coordinate. Outcomes measured from the modified version of the POWAR-TOTAL will include the Opal-based measures plus the time to complete the single-task trial, time to complete the dual-task trial, and cognitive task accuracy during the single-task (cognitive) and dual-task (physical + cognitive) trials (percentage of correct responses).

###### 5-10-5 Shuttle Run

**Figure 2:**
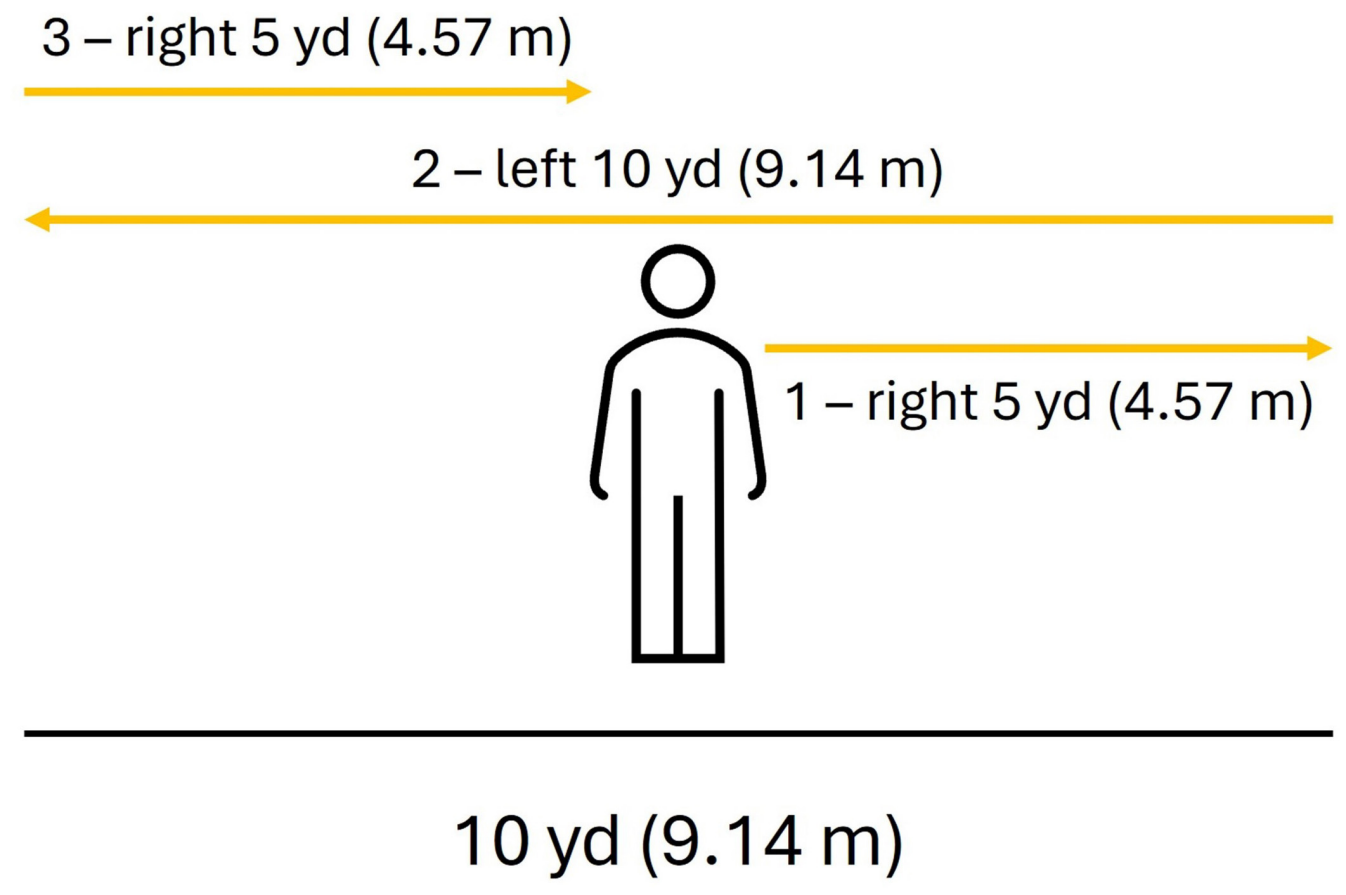
Schematic of the 5-10-5 Shuttle Run to the right. The service member begins the test in the middle and follows the path direction of the (yellow) arrows.

A part of the Ranger Athlete Warrior (RAW) assessment battery, participants will run five yards (4.57 m) to the right, turn and run 10 yards (9.14 m) to the left, then turn and run 5 yards (4.57 m) back through the starting position (Figure 2). This test of agility and quickness is then repeated in the opposite direction (to the left). Outcomes measured from the 5-10-5 Shuttle Run (45) will include the Opal-based measures plus the time to complete the trial to the right, and time to complete the trial to the left.

###### 300-yard Shuttle Run

**Figure 3:**
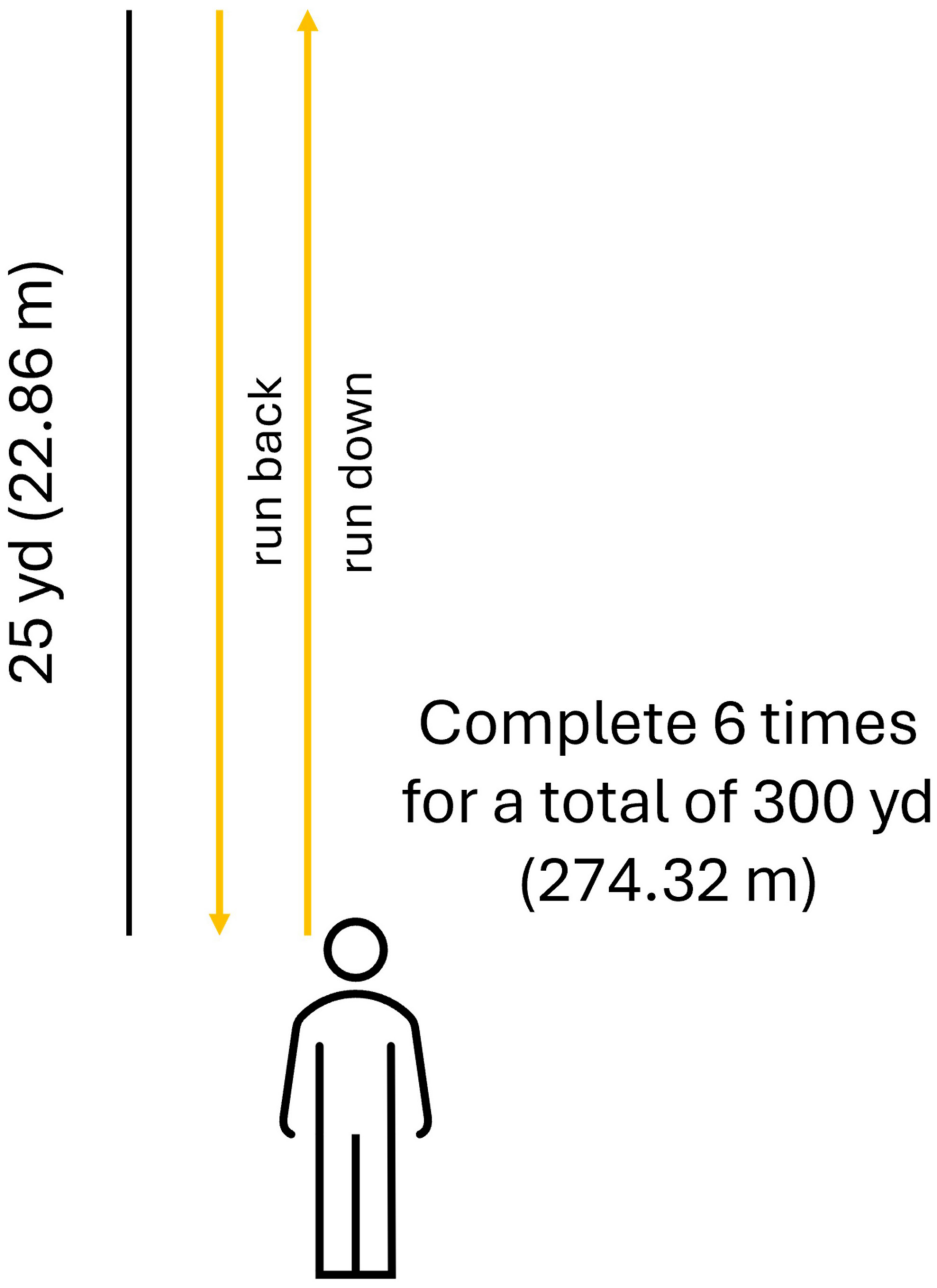
Schematic of the 300-yard Shuttle Run. The service member follows the path direction of the (yellow) arrows six times down and back.

As part of the RAW assessment battery, participants will run 25 yards (22.86 m), turn, and run 25 yards (22.86 m) back, completing this sequence six times for a total of 300 yards (274.32 meters; Figure 3). Participants must not use a circular path when making turns. The time taken to complete the single-task 300-yard Shuttle Run will be used to determine the interval for the cognitive-only single-task. Specifically, the participant is told an 8-digit grid coordinate, the stopwatch is started immediately after the final digit is spoken, the duration equivalent to the single-task 300-yard Shuttle Run (45, 46) completion time is waited, and then the participant is asked to recall the grid coordinate. Then, this cognitive task is combined with the physical task for a cognitive dual-task paradigm where the participant must remember an 8-digit grid coordinate; immediately upon completion of the 300-yard Shuttle Run the participant will be asked to recall the 8-digit grid coordinate. Outcomes measured from the 300-yard Shuttle Run will include the Opal-based measures plus the time to complete the single-task trial, time to complete the dual-task trial, and cognitive task accuracy during single-task (cognitive) and dual-task (physical + cognitive) trials (percentage of correct responses).

##### Resting-state functional MRI (rs-fMRI; Praxis group only)

Only participants in the Praxis group will undergo this assessment. With a Siemens 3T scanner (MAGNETOM Skyra Fit), anatomical and functional scans will be performed during each MRI session, alongside localizer scans. The acquisition parameters will follow established guidelines and standard methodologies to ensure comparability with previous studies. To this end, the team will closely mimic published protocols (14, 49), with the anatomical acquisition for co-registration intended to use magnetization-prepared rapid acquisition gradient echo (MPRAGE) imaging. The functional echo-planar imaging (EPI) sequence parameters will be selected to closely align with those used in prior research (14), including a repetition time (TR)/time to echo (TE) of 2,130 ms/30 ms, a field of view (FoV) of 220 mm x 220 mm, a voxel size of 3.4 mm x 3.4 mm x 3.4 mm, an axial slice number of 39, and a volume count of 245, with an expected rs-fMRI scan time of ~8.7 min. The rs-fMRI outcomes include functional connectivity (FC), regional homogeneity (ReHo), and fALFF, which will be analyzed to assess neurophysiological changes post-rehabilitation.

#### Four-week rehabilitation program

##### Control group

The Control group participants will complete 4 weeks of the SPaR Program without a HEP.

##### Praxis group

**Figure 4:**
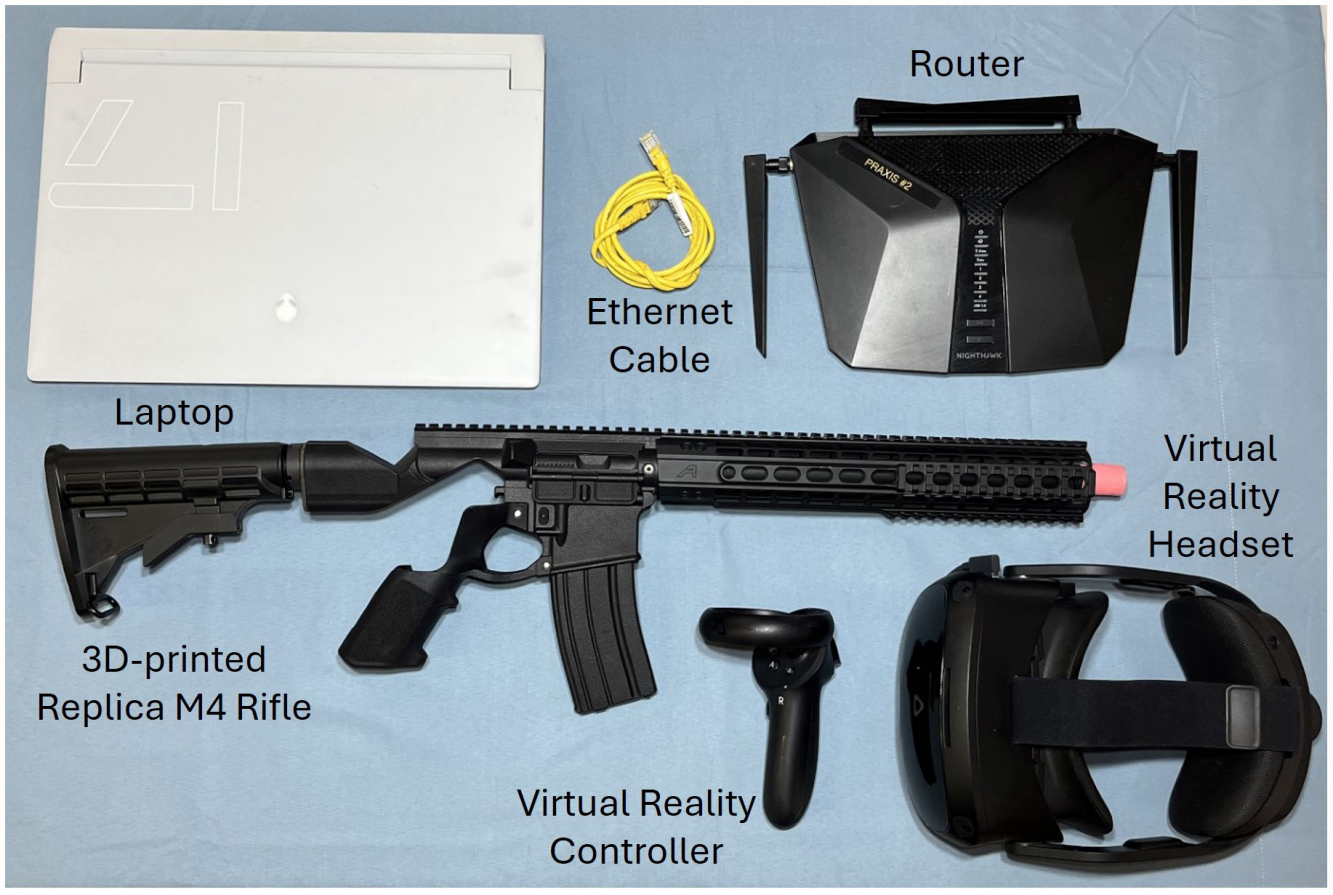
Praxis system components.

An innovative rehabilitation program has been designed to enhance function by integrating therapeutic elements into a VR-based platform, referred to as the Praxis system. The components of the Praxis system include a laptop, VR headset and controller, 3D-printed replica M4 rifle, router, and ethernet cable. The hardware details are summarized in Table 1 and Figure 4. The Praxis group participants will receive 4 weeks of Praxis-delivered augmented vestibular rehabilitation, which will be overseen by an Occupational Therapist. Each session will last 45 min daily, 5 days a week, and will be supplemented with a personalized HEP using the VestAid device (12) for 20 min a day, 5 days a week. Progression is individualized; the Occupational Therapist will adjust visual/cognitive load, duration, and movement parameters (e.g., head velocity) based on patient performance and symptoms, allowing treatment to be tailored to evolving functional abilities. Daily ratings for specific VOMS symptoms (headache, dizziness, nausea, and fogginess) will be collected pre- and post-exercise, rated on a 0–10 scale, to monitor symptoms throughout the rehabilitation.

**Table 1:** Praxis system components and specifications.

**Components**	**Specifications**
Alienware x17 R2 laptop and power adapter (Dell Technologies, Round Rock, TX)	Processor—12th Gen Intel Core i9-12900HK Graphics Card—NVIDIA RTX 3080 Ti RAM—32 GB
VIVE Focus 3 virtual reality headset with eye tracker and power adapter (HTC Corporation, Taiwan)	Virtual reality headset with eye tracker
VIVE Focus virtual reality controller and power adapter (HTC Corporation, Taiwan)	Only the right-hand controller is used in the replica M4 rifle
Replica M4 rifle (National Equipment Corporation (NECO), College Station, TX)	Approximate size and weight of a M4 Carbine rifle
Router and power adapter (NETGEAR, San Jose, CA)	NIGHTHAWK AX6 AX5400 6-Stream Wi-Fi router
Ethernet cable	Used to connect the laptop to the router

**Figure 5:**
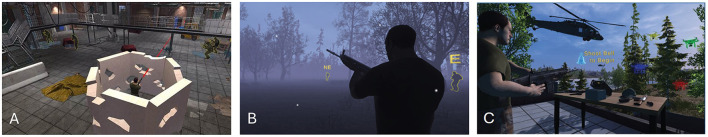
Praxis consists of three first-person-shooter serious games: **(A)** Barricade Wave Defense, **(B)** Directional Memorization, and **(C)** Stroop Target Shoot.

The Praxis-delivered augmented vestibular rehabilitation consists of three first-person-shooter serious games (Figures 5A–C). The Occupational Therapist can modify the visual complexity and cognitive demand of the games to adapt to patient needs. In Barricade Wave Defense (Figure 5A), patients shoot enemies from behind a barricade which requires them to move their heads and bodies. This game challenges attention, dynamic stability, agility, reaction time, gaze stability, and smooth pursuit. In Directional Memorization (Figure 5B), patients memorize direction orientation and then shoot enemies in response to directional cues. This game challenges spatial memory, attention, dynamic stability, gaze stability, and smooth pursuit. In Stroop Target Shoot (Figure 5C), patients memorize the location of colored drones and shoot those matching the color of the cue (vs. the named color). This game challenges spatial memory, attention, dynamic stability, response inhibition, reaction time, gaze stability, smooth pursuit, and saccades. The games provide a variety of outcome metrics, including response times, hit rates (accuracy), head velocity and rotation, and time on target (eye gaze).

#### Interim (weekly) assessments (Praxis group only)

At the end of each week, participants in the Praxis group will complete a GROC (30). They will also assess their current level of function on a scale from 0 to 100, with 100 representing optimal function (41).

#### Follow-up assessment

At the 4-week follow-up, all self-reported questionnaires (DHI, PSQI, GAD-7, PCL-M, VAAI-9, HIT-6, SBSOD, and fogginess rating) and the Readiness Assessment Battery (HAM-4-mTBI, modified version of the POWAR-TOTAL, 5-10-5 Shuttle Run, and 300-yard Shuttle Run) will be re-retested on all participants. The Praxis group will undergo another neurophysiological assessment (rs-fMRI) and will complete the SUS questionnaire (50) evaluating the Praxis system. Additionally, participants in the Praxis group will complete a final GROC (30) scale and reassess their current level of function on a scale from 0 to 100, with 100 representing optimal function (41).

#### Oura ring

Each participant will wear an Oura ring for the study duration. The Oura ring, equipped with a 3D accelerometer, will track activity levels and calculate daily energy expenditure using Metabolic Equivalents (METs). It will also monitor resting heart rate (an indicator of general health, wellness, and anxiety levels), heart rate variability (a potential marker of autonomic health, noting that some individuals show impairments following mTBI (51), which may improve post-rehabilitation), and the quantity and quality of sleep (sleep score, total sleep time, and wake-up time; as sleep is a key factor in recovery from mTBI (52)). Additionally, the activity score and inactive time will be recorded as markers of general health/wellness and to quantify activities conducted outside of the SPaR Program, which may act as confounding variables that need to be controlled for in statistical analyses.

**Figure 6:**
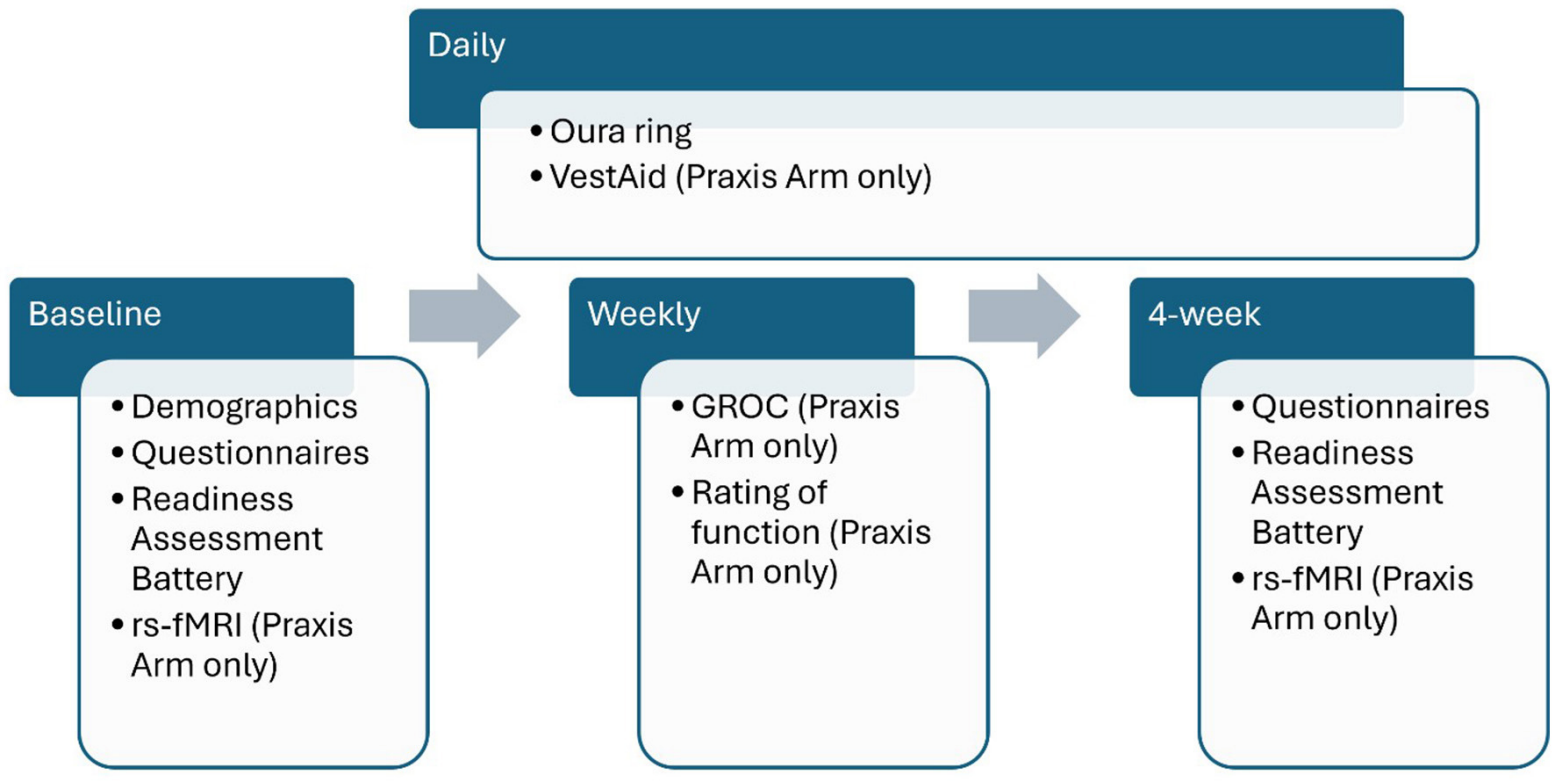
Schedule of data collection. GROC, Global Rating of Change; rs-fMRI, resting state functional magnetic resonance imaging.

The overall study procedures are reflected in Figure 6.

### Statistical/data analysis plan

#### Sample size and justification

The sample size of *n* = 15 for the Praxis group was determined based on practical feasibility, alignment with similar neurophysiological assessment studies (4, 14, 53), and funding constraints. Increasing the sample size beyond 15 offers minimal benefit in reducing confidence interval ranges (54), which are crucial for precision in effect size calculations for future studies.

Before performing any statistical analyses, we will assess all relevant statistical assumptions (e.g., normality, homogeneity of variance) to ensure appropriate test selection and the validity of results. Additionally, corrections for multiple comparisons will be applied to control the false discovery rate and minimize the risk of Type I error.

#### Descriptive statistics

Descriptive statistics, including percentages, means, and standard deviations will be used to describe the demographic characteristics of the sample, such as age, sex, height, weight, BMI, MOS/AOC, time in service, number and duration of deployments, number of self-reported and clinician-confirmed mTBI incidents and their causes, support structure (e.g., single, married), history of migraines/headaches, history of attention deficit hyperactivity disorder and/or learning disorders, and medical/surgical history. Differences within the sample will be analyzed using the chi-square test for proportions. Continuous variables will be analyzed using analysis of variance (ANOVA). The Oura ring data will be summarized as daily averages for each metric (e.g., METs, resting heart rate, heart rate variability, sleep score) and used as covariates in statistical analyses to control for individual variations in physical activity, sleep quality, and autonomic function. These covariates will help isolate the effects of the rehabilitation program on functional outcomes.

#### Usability and effectiveness of the Praxis system

The primary goal of this study is to evaluate the usability and effectiveness of the Praxis system. The usability of the Praxis system will be assessed using the SUS (50), a ten-item questionnaire with a five-point Likert scale, where “1” indicates strong disagreement and “5” indicates strong agreement. The SUS scores will be calculated at both the individual level and the group level. Success will be measured by user satisfaction, with a target of an average SUS score of 68 or higher at the group level. A multi-level factorial analysis will be conducted to identify different factor structures for each first-person-shooter serious game. Cronbach's alpha will be used to measure the internal consistency of the 10 SUS items. For effectiveness, descriptive statistics, including means, standard deviations, and 95% confidence intervals will be calculated for the behavioral outcomes. Paired samples *t*-tests will be used to compare DHI, PSQI, GAD-7, PCL-M, VAAI-9, HIT-6, and SBSOD variables before and after the 4-week rehabilitation program. In addition to usability and effectiveness, success will be measured by the acceptability of the rehabilitation protocol, with a target of at least 80% of the Praxis group completing all prescribed rehabilitation sessions.

Data from participants who deviate significantly from the protocol (< 50% adherence) or have missing data will be excluded. The Shapiro-Wilk test will be used to assess the normality of the data. For non-normal continuous or ordinal data, median and interquartile ranges will be calculated, and percentages will be reported for categorical data. For non-normal data, Poisson regression will be applied. Linear regression will be employed to study and adjust for baseline imbalances between groups, with results reported as adjusted mean differences between the groups and their confidence intervals. A significance level of *p* < 0.05 will be used to determine meaningful differences.

#### Neuroimaging changes following Praxis treatment

The neuroimaging will involve structural (sMRI) and rs-fMRI data acquired from a Siemens 3T scanner (MAGNETOM Skyra Fit) at Brooke Army Medical Center. Key imaging parameters for sMRI are TR = 2,530 ms, TE = 2.6 ms, FA = 7o, FOV=256 mm × 256 mm, Matrix = 256 × 256, Sagittal Slices = 176, Thickness = 1 mm; and for rs-fMRI, TR = 2,130 ms, TE = 30 ms, FA = 85o, FOV=220 mm × 220 mm, Matrix = 64 × 64, Axial Slices = 39; Thickness = 3.4 mm, Measurement = 245.

Using our data analysis pipeline implemented with the “AFNI” software package (afni.nimh.nih.gov), the analysis at the individual level will go through eight processing steps of (i) outlier detection, (ii) despiking, (iii) slice timing correction, (iv) volume registration, (v) template normalization into the MNI space (55), (vi) image segmentation for gray matter, white matter, and cerebral spinal fluid (CSF), (vii) nuisance signal (head motion, white matter, and CSF) regression, and (viii) spatial smoothing (FWHM = 5 mm). Subsequently, the analysis of rs-fMRI will evaluate differences in the (i) FC (56), (ii) ReHo (57), and (iii) fALFF (58) between baseline and post-rehabilitation. While the ReHo and fALFF will be evaluated across the whole brain, FC will be evaluated in the vestibular visual network (59). Voxel-wise values of FC, ReHo, and fALFF will be derived individually, and linear-mixed-effect (LME) modeling (60) will be performed, using imaging times (pre- vs. post-rehabilitation) as the variable of interest and head motion as the covariate. The rehabilitation effect revealed by this analysis will be corrected for multiple comparison by a Monte Carlo simulation (61).

#### Relationships between neuroimaging changes and behavioral outcomes

Correlation analyses will be performed to examine the relationships between rs-fMRI findings and behavioral outcomes (from questionnaires and the Readiness Assessment Battery tasks: HAM-4-mTBI, modified version of the POWAR-TOTAL, 5-10-5 Shuttle Run, and 300-yard Shuttle Run), as well as clinical characteristics, with a significance threshold set at *p* < 0.05.

By comparing the behavioral outcomes from questionnaires and the Readiness Assessment Battery between baseline and post-rehabilitation, this study aims to identify any significant changes occurring between these assessment points. To compare between-group differences in behavioral outcomes, improvements during the 4-week intervention will be calculated by subtracting the baseline value (the first visit) from the post-rehabilitation value. A general linear model will be used to assess between-group differences in primary and secondary outcomes. Estimated marginal means, standard errors (SEs), 95% confidence intervals, and effect sizes will be reported for the outcomes derived from the general linear model. Effect sizes will be interpreted as small (0.01), medium (0.06), and large (0.14) (62). The Minimum Clinically Important Difference (MCID) will be calculated for all outcomes using the equation MCID = SD ^*^ 0.2.

#### Praxis scores as mediators of neuroimaging changes and behavioral outcomes

A mediation analysis will be conducted to explore whether Praxis scores (e.g., response times, accuracy, head velocity) mediate the relationship between rs-fMRI outcomes and behavioral outcomes. For all analyses, we will use the Praxis metrics that account for and remove the influence of game parameters (i.e., tailored treatments) selected by the Occupational Therapist. This analysis will follow a four-step regression-based approach:

1. Establish a significant association between rs-fMRI outcomes and behavioral outcomes.
2. Verify a significant association between rs-fMRI outcomes and Praxis scores.
3. Demonstrate that Praxis scores predict behavioral outcomes while controlling for rs-fMRI outcomes.
4. Compare the direct effect of rs-fMRI outcomes on behavioral outcomes with its effect in the presence of Praxis scores. Bootstrapping will confirm the significance of the mediation effect.

By following this method, we will pinpoint the Praxis scores that significantly mediate the relationship between rs-fMRI outcomes (body structure and function impairments) and behavioral outcomes. Individual factors, such as demographic and personal factors, will also be considered. The findings will help formulate hypotheses and guide the design of future clinical studies to explore functional performance thresholds.

#### Praxis performance as a marker of recovery (readiness for return to duty)

This study may provide insight into reasonable thresholds for RTD decisions based on behavioral outcomes and provide insights for future research on the role of Praxis-delivered augmented vestibular rehabilitation in recovery from mTBI. Accordingly, the team will conduct the following statistical analyses.

We propose that participants' performance on the Praxis system can serve as an indicator of their recovery from mTBI. The team aims to test this hypothesis by comparing longitudinal rehabilitation compliance scores with behavioral outcomes and self-reported symptom severity. At a broader population level, we anticipate that participants who exhibit the most significant increases in Praxis scores (metrics that account for and remove the influence of game parameters selected by the Occupational Therapist) will also show the most substantial improvements in their performance on the Readiness Assessment Battery and in self-reported symptom severity, as measured before and after the 4-week intervention. To validate this, we will use standard statistical methods to compare weekly Praxis performance metrics (calculated as the average outcomes for each session within a week). Additionally, we expect that day-to-day participation in Praxis will correlate with the overall recovery trajectory from mTBI. To examine this, we will compare longitudinal data collected from Praxis, specifically focusing on (A) daily Praxis performance metrics and (B) daily pre- and post-exercise VOMS symptom scores (38). To test the hypothesis that these measures are correlated within a session, we will employ methods such as first-differences regression to correct for directional biases and identify correlations in these longitudinal data points.

We additionally propose that the Praxis group's behavioral outcomes following the 4-week intervention will meet or exceed those of the Control group, which serves as a normative reference for self-reported symptom severity and functional performance on military-specific tasks in this unique population. To examine this, we will compare between-group differences at the 4-week timepoint. The Control group will establish the presumed reasonable thresholds for RTD decisions. We will report the number of individuals in the Praxis group who meet or exceed these thresholds. The team plans to investigate the relationship between participants' Praxis scores, behavioral outcomes, and the underlying neurophysiological changes throughout recovery from mTBI, using neuroimaging biomarkers identified in prior studies (4, 14, 18). Specifically, we will test the hypothesis that improvements in behavioral outcomes are associated with enhanced abilities as measured by Praxis scores, as well as functional changes observed through rs-fMRI. We extend this hypothesis by suggesting that if performance on Praxis exercises predicts improvements in behavioral outcomes, it does so in a manner that could be linked to detectable changes in the underlying neurophysiology. To test this, the team will conduct a mediation analysis, which is a regression-based method to assess how error propagates through multiple related variables by evaluating the likelihood of alternative models that include primary and mediating variables.

A potential challenge of our study design is the need for significant correlations between the measures being tested to justify a mediation analysis. Given that our study is the first of its kind, it is not guaranteed that the performance on Praxis exercises and the neurophysiological measures will correlate with improvements in behavioral outcomes. To mitigate this risk, the team plans to conduct a series of secondary analyses to derive as many insights as possible from this pilot study. First, the team will evaluate the functional neuroimaging data to test hypotheses established in previous studies. Second, the team will explore whether there are neurophysiological features related to Praxis performance that do not manifest in the behavioral outcomes.

## Discussion

After mTBI, SMs often have vestibular and ocular deficits that impair their ability to perform critical tasks essential for military readiness and operational effectiveness (8). While overt balance and mobility deficits typically resolve within a few days (63), abnormalities can persist and can be missed in routine clinical assessments. These lingering deficits have been identified using advanced instrumentation and analytical methods (64–72). These residual deficits, particularly in complex, real-world tasks, can compromise the overall performance and safety of SMs in demanding operational environments (3, 5). Despite a growing recognition of the importance of addressing vestibular dysfunction in post-mTBI rehabilitation, there remains a significant gap in understanding the best strategies to mitigate these impairments, especially when it comes to rehabilitation dosage.

Current rehabilitation techniques are not consistently supported by strong evidence, and the VA/DoD Clinical Practice Guidelines (CPGs) do not provide clear recommendations for optimal rehabilitation dosages or strategies (25). Access to specialized care may be limited at military treatment facilities without dedicated resources, such as Intrepid Spirit Centers. Traditional assessments, which often rely on single-task evaluations, may lack the sensitivity to detect subtle, lingering deficits (73, 74). Furthermore, current RTD decisions typically rely on self-reported symptoms and standardized physical and cognitive tests, which may not accurately reflect the functional demands of military activities (6–9), limiting their ability to drive tangible improvements in operational performance. This ambiguity presents a clear need for more comprehensive, objective, and ecologically valid rehabilitation interventions that can better capture and address the multifaceted impairments experienced by SMs with mTBI.

The Praxis study aims to fill this gap by investigating the potential benefits of a novel VR-based rehabilitation system designed specifically for SMs with mTBI-related vestibular complaints. By integrating real-world, military-relevant tasks within a multisensory framework, the Praxis system provides an engaging rehabilitation tool with face validity that simulates some of the sensory, motor, and cognitive demands SMs face in operational settings. Moreover, increasing the complexity of rehabilitation tasks, such as incorporating dual-task elements, may reveal subtle abnormalities that traditional, simpler assessments fail to detect (73–76). This could improve clinical decision making, particularly for RTD assessments, by providing more nuanced and objective measures of functional recovery (77).

Wearable sensors embedded in the Praxis system allow for the continuous monitoring of complex motor tasks, such as turning, which is an ecologically relevant action frequently performed by SMs (22). Recent developments in wearable technology have made it possible to conduct objective and sensitive evaluations of subtle motor impairments during routine clinical assessments (78–81). These objective assessments, free from the limitations of arbitrary ceiling effects, enable precise tracking of rehabilitation progress and functional outcomes (77). If the findings from this study demonstrate that the Praxis system is effective, it could offer new evidence for revising the VA/DoD CPGs to include VR- and sensor-based technologies as standard components of vestibular rehabilitation after mTBI.

Moreover, the Praxis system could lead to more consistent and evidence-based decision making regarding RTD determinations by providing rehabilitation protocols that are not only responsive to rehabilitation but also directly associated with performance in real-world tasks. Should the Praxis system prove effective, it could motivate widespread adoption of such technologies in post-mTBI rehabilitation, potentially guiding future policy updates and clinical protocols.

Despite these promising directions, several limitations should be acknowledged. First, the small sample size restricts the generalizability of our findings and necessitates caution in extrapolating results to a larger or more diverse population. Second, this single-site study focuses on a specialized military cohort, which may not capture the full spectrum of clinical presentations seen in broader populations with mTBI. Third, the non-randomized study design limits our ability to infer causal relationships. Finally, although the Praxis group will receive the standardized SPaR Program plus the novel VR-based intervention, other concurrent treatments or variations in standard care could influence rehabilitation outcomes. Future research should address these limitations by employing larger, more diverse samples, multicenter trials, and randomized controlled designs to strengthen the evidence base for VR-based vestibular rehabilitation in military and civilian populations.

Ultimately, this pilot study has the potential to drive meaningful changes in how vestibular dysfunction post-mTBI is assessed and treated, particularly within the military population. By providing insights into optimal rehabilitation dosages and the use of technology-assisted interventions, the Praxis study could pave the way for more extensive trials and broader adoption of objective, real-world rehabilitation strategies. This, in turn, may help ensure that SMs recover more fully and are better prepared to meet the physical and cognitive demands of their warfighter duties.
